# Larval secretions of parasitoid wasps are new effectors that impair host immune defences

**DOI:** 10.1007/s44297-023-00011-y

**Published:** 2023-11-15

**Authors:** Lan Pang, Zhi Dong, Zhiguo Liu, Ting Feng, Wenqi Shi, Yueqi Lu, Yifeng Sheng, Jiani Chen, Xueying Guan, Xuexin Chen, Jianhua Huang

**Affiliations:** 1 Institute of Insect Sciences, Ministry of Agriculture Key Lab of Molecular Biology of Crop Pathogens and Insect Pests, Zhejiang University, Hangzhou, China; 2 Key Laboratory of Biology of Crop Pathogens and Insects of Zhejiang Province, Zhejiang University, Hangzhou, China; 3 Zhejiang Provincial Key Laboratory of Crop Genetic Resources, Institute of Crop Science, Plant Precision Breeding Academy, College of Agriculture and Biotechnology, Zhejiang University, Hangzhou, China; 4 Guangdong Laboratory for Lingnan Modern Agriculture, Guangzhou, China

**Keywords:** Parasitoid wasps, Larval secretions, Host immunity, Serpin, Metalloprotease

## Abstract

Parasitoid wasps belong to a remarkably successful group of biological control agents. To ensure effective parasitism, parasitoid wasps have evolved various effectors to modulate host immune responses, such as venom, PDVs and teratocytes. However, little information is available for wasp larval secretions. Here, we applied the *Drosophila* parasitoids *Leptopilina boulardi* and *L. heterotoma* as a study model to investigate the larval secreted components and the potential molecules manipulating host immune responses. We obtained 388 and 30 larval secreted proteins from *L. boulardi* and *L. heterotoma*, respectively. These results were consistent with the fact that *L. boulardi* larvae encounter more severe immune challenges in comparison with *L. heterotoma*. We further revealed that parasitoid wasps have evolved specialized secreted proteins to inhibit host melanization responses via serpins, as well as a group of metalloproteases may contribute to impairing host encapsulation responses. Our findings provide the evidence that larval secretions of parasitoid wasps are new effectors and further uncover their potential roles in manipulating host immune responses.

**Supplementary Information:**

The online version contains supplementary material available at 10.1007/s44297-023-00011-y.

## Introduction

Parasitoid wasps, also known as parasitoids, are common insect species in Hymenoptera. In general, about 75% of Hymenoptera species are parasitoid wasps, comprising an estimated number of 150,000 to 600,000 species [1–3]. Female parasitoids often lay eggs in (endoparasitoids) or on (ectoparasitoids) their associated arthropod hosts, while the hatched wasp larvae rely on gradually consuming the hosts for development and survival [2, 4]. Since the successful parasitism often results in the death of hosts, parasitoid wasps act as natural enemies and biocontrol factors in controlling host populations within ecosystems [5, 6]. Moreover, the hosts and parasitoid wasps usually engage in an arms race for survival: whereas the hosts tend to increase their resistance, the parasitoids evolve to improve their success [7, 8].

The hosts have developed efficient and powerful innate immune responses to defend against the infection by parasitoid wasps, which are classically divided into two main parts: humoral and cellular immune responses [9, 10]. Humoral responses include the production of antimicrobial peptides and reactive intermediates of oxygen or nitrogen, and the activation of prophenoloxidase (PPO) to phenoloxidase (PO) that contributes to melanization [10, 11]. Cellular responses refer to hemocyte-mediated immune defenses like phagocytosis, nodulation and encapsulation [9, 10]. Melanization and encapsulation are common phenomena in infected hosts that help to kill parasitoid wasps, and the detailed processes have been well studied in *Drosophila*-parasitoid systems. In *Drosophila* larvae, three types of mature hemocytes are recognized including plasmatocytes, lamellocytes and crystal cells. First, plasmatocytes recognize and attach to the invading wasp eggs, and they spread around the surface of the wasp eggs forming the first layer of the capsule. Then, lamellocytes formed in response to wasp infection surrounded on the plasmatocyte-coated eggs. The accumulation of lamellocytes around the eggs is accompanied by flattening of the blood cell layers, further isolating the parasitoid eggs. Finally, crystal cells and lamellocytes release phenoloxidase, which causes in melanization of the capsule, leading to the death of wasps [12–15].

In order to overcome the host immune responses for successful parasitization, parasitoids have developed different strategies by using of an arsenal of effectors, which are the components the parasitoids inject or release into the host during parasitization that play vital roles in facilitating successful parasitism, such as venom, polydnavirus (PDVs), and teratocytes [2, 16–18]. Maternally transmitted effectors including venom and PDVs are injected into the host hemocoel during oviposition. Venom is a complex cocktail of proteins (usually enzymes) carried by almost all parasitoid wasps and reported to play important roles in suppressing host immune responses in some parasitoid species including *Leptopilina heterotoma*, *Nasonia vitripennis*, *Pteromalus puparum* and *Pimpla hypochondriaca* [19–22]. PDVs are a group of large double-stranded DNA viruses that are obligatory symbionts of parasitoid wasps in the Ichneumonidae and Braconidae families [23, 24]. PDVs can integrate into host genomes and subsequently express the virulence genes to manipulate the host physiology. It has been reported that expression of these virulence genes can suppress host immune responses, preventing the parasitoids from melanization and encapsulation [25–27]. Teratocytes are non-maternal effectors that dissociate from the embryonic membrane during the wasp egg hatching. Teratocytes have been found to provide essential nutrition for the development of wasp larvae, and they are mainly present in a few number of parasitoid wasp species (e.g., *Microplitis croceipes*, *Perilitus coccinellae* and *Aphidius ervi*) [28–30]. Although the functions of venom, PDVs and teratocytes have been widely investigated, the studies aiming at finding some other effectors are still ongoing [31–33]. Recently, several studies have revealed that herbivorous insects contain active molecules in their oral secretions, which help interfere with plant defense during herbivory [34, 35]. These interesting findings allow us to propose a hypothesis that the parasitoid larvae might also produce effectors like those from oral secretions when they consuming their hosts.

The parasitoid wasps in the genus *Leptopolina* (Figitidae) are well known for parasitizing *Drosophila* species [36]. *L. heterotoma* (Lh) and *L. boulardi* (Lb) are two endoparasitoids that prefer to infect second instar *Drosophila melanogaster* larvae, but have different parasitic characteristics. First, Lh can successfully parasitize a number of species across the *Drosophila* genus, whereas Lb is only specially adapted to *D. melanogaster* and its close relatives. Second, Lh employs an active immune suppression strategy, which destroys host hemocytes to inhibit melanization and encapsulation, thereby facilitating successful parasitism. In contrast, Lb employs a passive immune evasion strategy, with wasp eggs becoming attached to host internal tissues, providing physical protection against complete encapsulation by host hemocytes [22, 37–39]. As such, Lh and Lb provide excellent models for exploring the genetic basis of parasitoid-host coevolution. In this study, we used Lh and Lb to answer the following two questions: (1) Do the parasitoids utilize larval secretions as the parasitic effectors to regulate host immune responses? (2) Are their functional activities diverse in different parasitoids?

## Results

### The host cellular immune responses post Lb and Lh parasitism

**Fig. 1:**
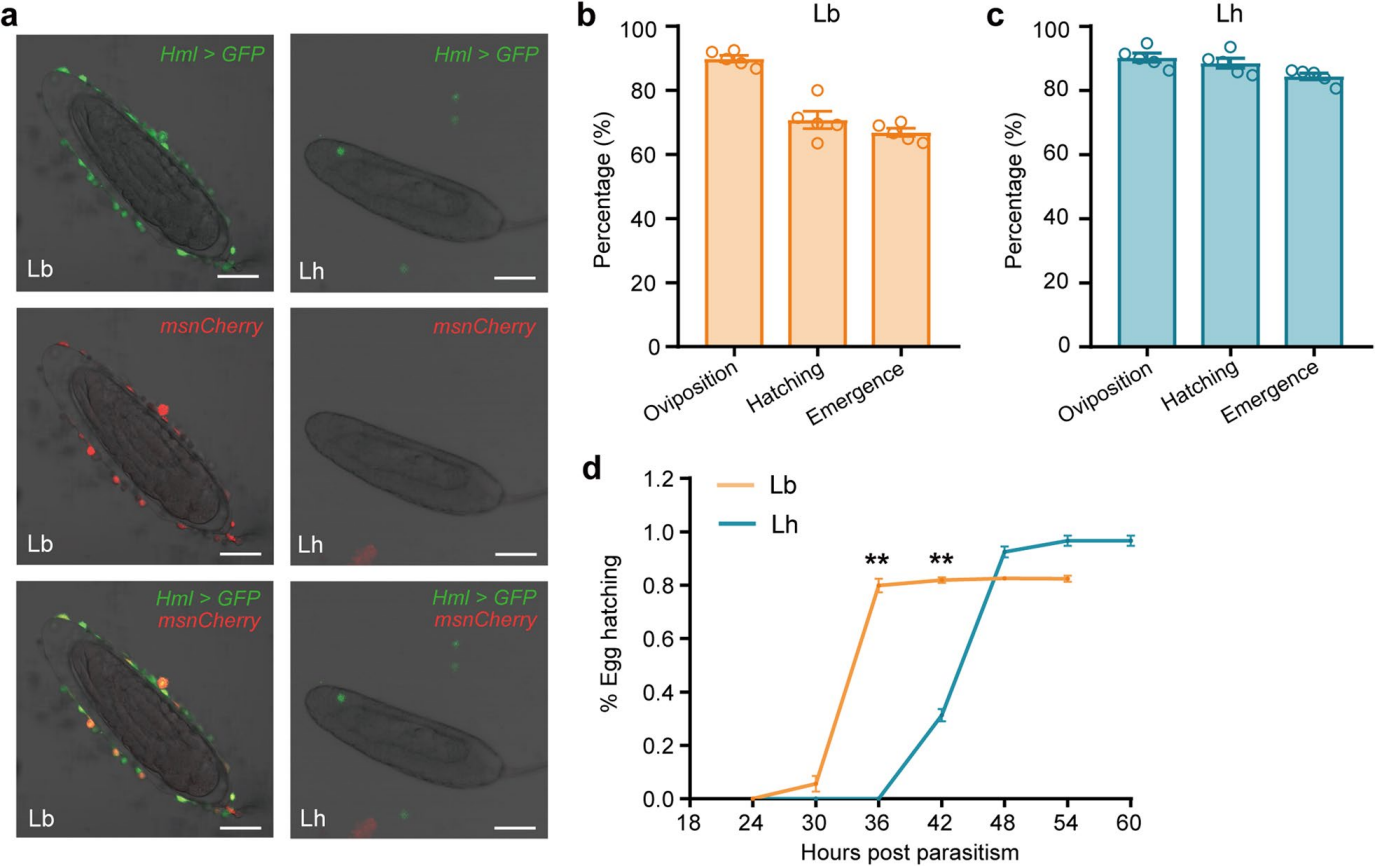
Host encapsulation response after Lb and Lh parasitism. **a** The host hemocytes adhering to the surface of wasp eggs at 24 h post parasitism. The circulating hemocytes (*Hml* > *GFP*) and lamellocytes (*msnCherry*) were visible on the Lb eggs, while no hemocytes attached to the freely floating eggs of Lh. Scale bars: 50 μm. **b** and **c** The oviposition rate, hatching rate and emergence rate of Lb (**b**) and Lh (**c**). Five independent biological replicates were performed and at least 300 insects were examined for each treatment. Data represented the mean ± SEM. **d** Wasp egg hatching rate of Lb and Lh was observed every 6 h after parasitism. Five independent biological replicates were performed and at least 260 insects were examined for each treatment. Data represented the mean ± SEM. The significance of the mean of the 36 h and 42 h egg hatching rates was analysed by the Mann‒Whitney U test (**p < 0.01)

To investigate the immune responses post Lb (Evasion strategy) and Lh (Suppression strategy) oviposition, we visualized the host hemocytes using two previously developed reporters. One is the hemocyte-specific GAL4 and fluorescent enhancer-reporter fusion construct (*Hml-GAL4, UAS-2* × *EGFP*; here called *Hml* > *GFP*), which labels all circulating hemocytes (e.g. plasmatocytes, crystal cells, and lamellocytes) [39]. Another is the enhancer-reporter construct *MSNF9MO-mCherry* (*msnCherry*), which is specific for lamellocytes [39, 40]. We then dissected wasp eggs at 24 h post oviposition, and examined the host encapsulation reaction. In Lb infected hosts, the hemocytes were observed attached and spread on wasp eggs, while there were few, if any, hemocytes adhered to Lh eggs (Fig. 1a). Our results confirm the previous reports that the encapsulation response is obvious in Lb-parasitized hosts, but not in Lh-parasitized hosts [22, 39].

We further found that the hatching rates (number of hosts containing hatched larvae/number of total hosts × 100%) of Lb and Lh wasp eggs were all very high, with 71% for Lb and 89% for Lh, respectively (Fig. 1b and c). How could Lb eggs escape the host encapsulation response? We monitored the hatching time of Lb and Lh wasp eggs, respectively. The Lb eggs initiated hatching at 24 h post oviposition, and almost all eggs successfully hatched into larvae at 36 h. Different with Lb, Lh eggs did not hatch yet at 36 h, and almost all eggs hatched between 36–48 h post oviposition (Fig. 1d). These results show that Lb takes a much shorter time to complete its embryonic development than Lh. In addition to the well-known “attached” strategy of Lb eggs attaching to host internal tissues to escape the host encapsulation [22, 41], our findings also raise the possibility that Lb has evolved a “quick-development” adaption to escape the complete encapsulation and survive.

### Lb larvae suffer higher cellular immune challenges than Lh

**Fig. 2:**
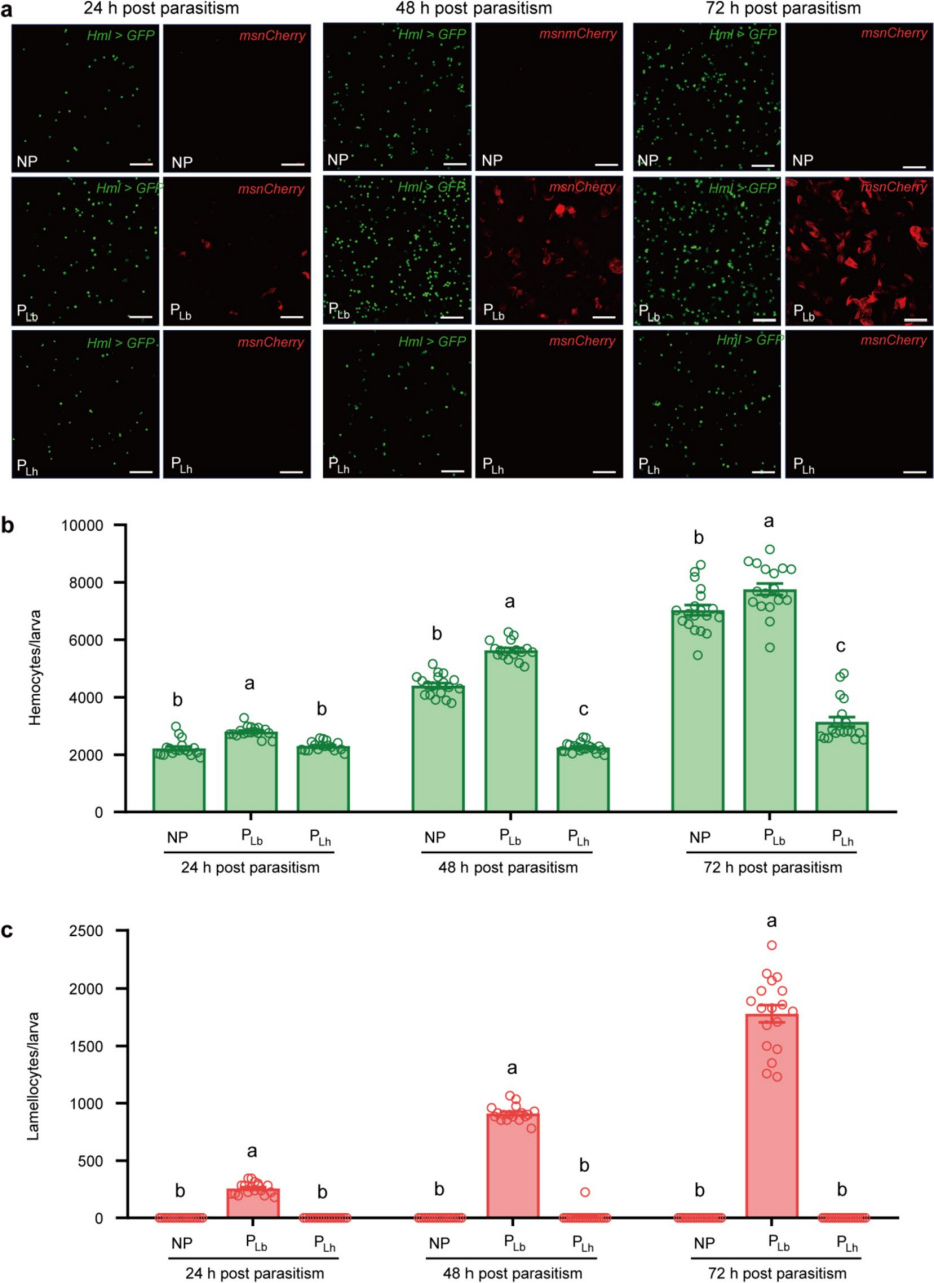
Lb larvae encounter a much higher host immune response. **a** Observation of circulating hemocytes and lamellocytes in host larvae at 24 h, 48 h and 72 h post parasitism. Scale bars: 50 μm. **b** The amount of circulating hemocytes at 24 h, 48 h and 72 h post parasitism. At least fifteen independent biological replicates were performed and shown as dots. Data represented the mean ± SEM. Significance was analysed by one-way ANOVA with Sidak's multiple comparisons test. Different letters indicate statistically significant differences (*P* < 0.05). (24 h, *F* = 37.46, *df* = 2, 50, *P* < 0.0001; 48 h, *F* = 621.2, *df* = 2, 52, *P* < 0.0001; 72 h, *F* = 186.7, *df* = 2, 53, *P* < 0.0001). **c** The amount of lamellocytes at 24 h, 48 h and 72 h post parasitism. At least fifteen independent biological replicates were performed and shown as dots. Data represented the mean ± SEM. Significance was analysed by the Kruskal–Wallis test with Dunn's multiple comparisons test. Different letters indicate statistically significant differences (*P* < 0.05). (24 h, *Kruskal–Wallis statistic* = 49.19, *P* < 0.0001; 48 h, *Kruskal–Wallis statistic* = 49.83, *P* < 0.0001; 72 h, *Kruskal–Wallis statistic* = 52.36, *P* < 0.0001). NP, nonparasitized host; P_Lb_, Lb parasitized host; P_Lh_, Lh parasitized host

With the help of *Hml* > *GFP* and *msnCherry*, we then counted the number of host circulating hemocytes at different time points post oviposition including 24 h, 48 h, and 72 h. Notably, both Lb (Evasion strategy) and Lh (Suppression strategy) developed to the larval stage at 48 h and thereafter post oviposition. Consistent with previous observations, the total amount of circulating hemocytes was increased during *D. melanogaster* development, and no lamellocytes were found in unparasitized host larvae [39]. However, large quantities of hemocytes and lamellocytes were produced at 48 h and 72 h after infection by Lb (Fig. 2). In contrast, no induction of lamellocytes was observed in Lh parasitized hosts, as compared with unparasitized hosts. The total number of circulating hemocytes in Lh parasitized hosts was significantly decreased compared to that in unparasitized and Lb parasitized hosts (Fig. 2), confirming the previous findings that Lh parasitism causes rapid lysis of host hemocytes [22, 39].

We next tested the parasitic efficiency of the two *Leptopilina* wasps, and found that the wasp offspring emergence rates (number of emerged wasps/number of total hosts × 100%) of Lb and Lh were 67% and 84%, respectively. Most importantly, the wasp offspring emergence rate was comparable to the hatching rate for each parasitoid species (Fig. 1b and c). Nearly all successfully hatched Lb larvae were able to develop into adults despite continuously elevated host immune challenges (Figs. 1 and 2). These results led us to speculate that *Leptopilina* larvae might manipulate host immune responses to survive through their secretions, and the manipulation ability of Lb secretions is much stronger than that of Lh.

### Lb larval secretions impair host immune responses

**Fig. 3:**
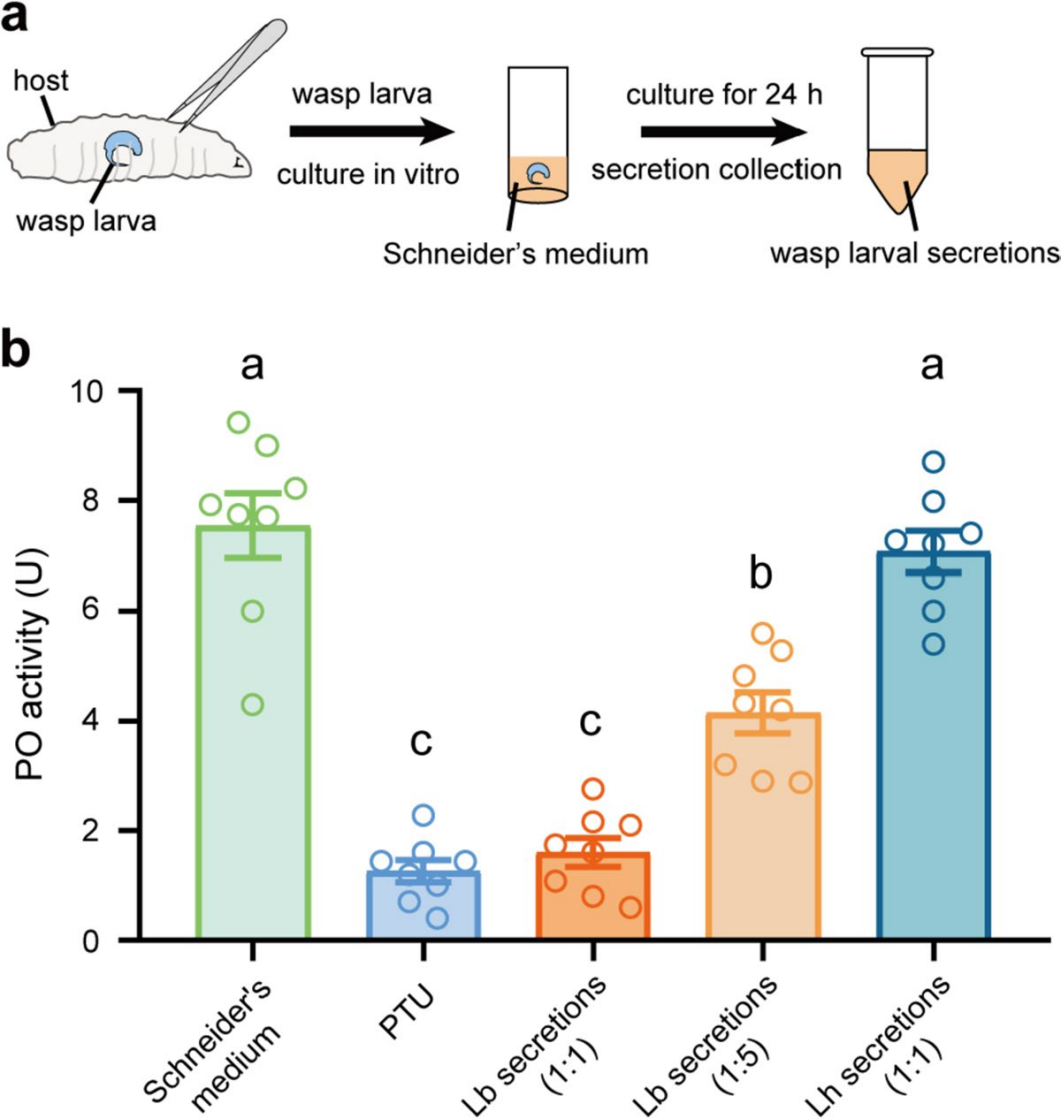
Lb larval secretions inhibit host melanization response. **a** Schematic diagram of collecting wasp larval secretions. **b** PO activity of host hemolymph after incubation with Schneider's medium (negative control), PTU (positive control), Lb secretions (1:1), Lb secretions (1:5), and Lh secretions (1:1). Eight independent biological replicates were performed. Data represented the mean ± SEM. Significance was analysed by one-way ANOVA with Sidak's multiple comparisons test. Different letters indicate statistically significant differences (*P* < 0.05). (*F* = 59.02, *df* = 4, 35, *P* < 0.0001)

To test whether wasp larvae could manipulate host immunity through their secretions, both Lb and Lh were dissected from parasitized hosts and cultured in vitro for 24 h to produce secretions (Fig. 3a). We collected and compared the protein concentration of the two wasp larval secretions, and found that the concentration of Lb secretions was much higher than that of Lh (Online Resource 1, Fig. S1), indicating that Lb produces more secretions to protect themselves.

The activity of PO which catalyzes the oxidation of phenols to quinones to trigger the melanization response of host hemolymph was measured to investigate the effect of larval secretions on the host melanization response [10, 11, 42]. Different dilutions of the Lb larval secretions (1:1 or 1:5) were added to host hemolymph, and each individual dose significantly inhibited the PO activity compared to the control. Moreover, the PO activity of secretions-treated host hemolymph was markedly lower at a 1:1 dilution than at a 1:5 dilution (Fig. 3b). As expected, Lh larval secretions showed no effect on host PO activity (Fig. 3b). Thus, our results suggest that Lb larval secretions reduce the host melanization response.

**Fig. 4:**
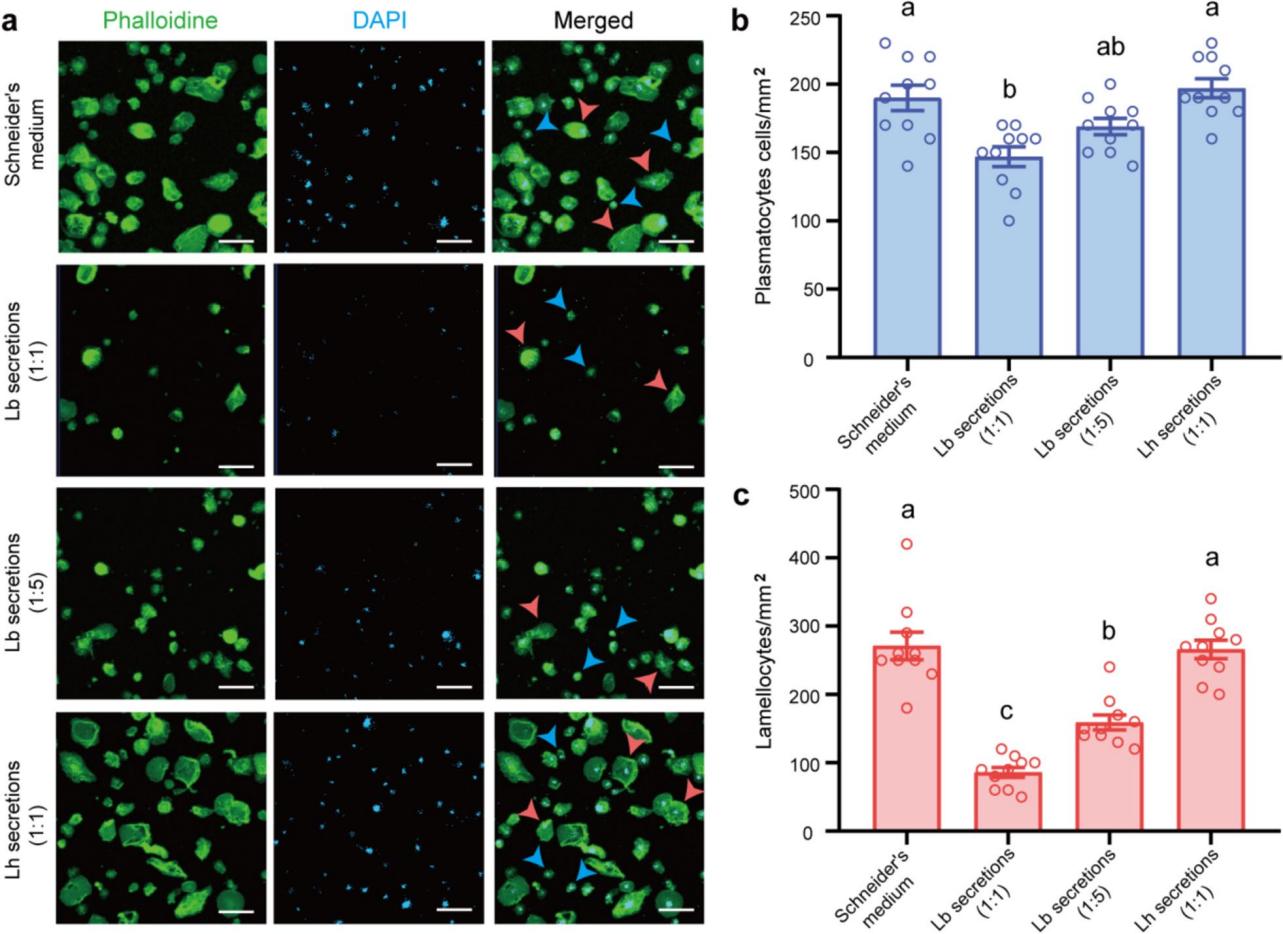
Lb larval secretions impair the adhesion of host hemocytes. **a** Immunofluorescence detection of hemocyte adhesion after treatment with Schneider's medium (negative control), Lb secretions (1:1), Lb secretions (1:5), and Lh secretions (1:1). The nucleus was stained with DAPI (blue), and the cytoskeleton was labelled with phalloidin 488 (green). The blue and green arrows indicated the representative plasmatocytes and the lamellocytes, respectively. Scale bars: 50 μm. **b** and **c** The number of adherent plasmatocytes (**b**) and lamellocytes (**c**). Ten independent biological replicates were performed. Data represented the mean ± SEM. Significance was analysed by one-way ANOVA with Sidak's multiple comparisons test. Different letters indicate statistically significant differences (*P* < 0.05). (**b*, F*** = 9.021, *df* = 3, 36, *P* = 0.0001; **c*, F*** = 41.81, *df* = 3, 36, *P* < 0.0001)

The adhesion ability of host hemocytes was also measured to investigate the effect of larval secretions on the host encapsulation response, as cell adhesion is the necessary step to initiate the encapsulation [9]. *HOP*^*Tum−1*^ is a mutation fly line that spontaneously produces lamellocytes [43, 44]. Therefore, *HOP*^*Tum−1*^ larvae contain both plasmatocytes and lamellocytes, which mimic the hemocytes in wasp infected hosts. The hemocytes of *HOP*^*Tum−1*^ larvae were dissected and allowed to adhere to the glass surface of a microscope slide. After 30 min, the numbers of adhesive cells were counted after different treatments. In comparison to the control, different dilutions of Lb larval secretions (1:1 or 1:5) significantly reduced the numbers of plasmatocytes and lamellocytes on the slide. Moreover, the cell numbers after Lb secretions treatment at a 1:1 dilution were markedly lower than those treated at a 1:5 dilution. As expected, Lh larval secretions showed no effect on cell adhesion activity (Fig. 4). Thus, our results suggest that Lb larval secretions might impair the host encapsulation response via reducing the adhesion ability of host hemocytes.

### Identification of the larval secretions catalogue of Lb and Lh

**Fig. 5:**
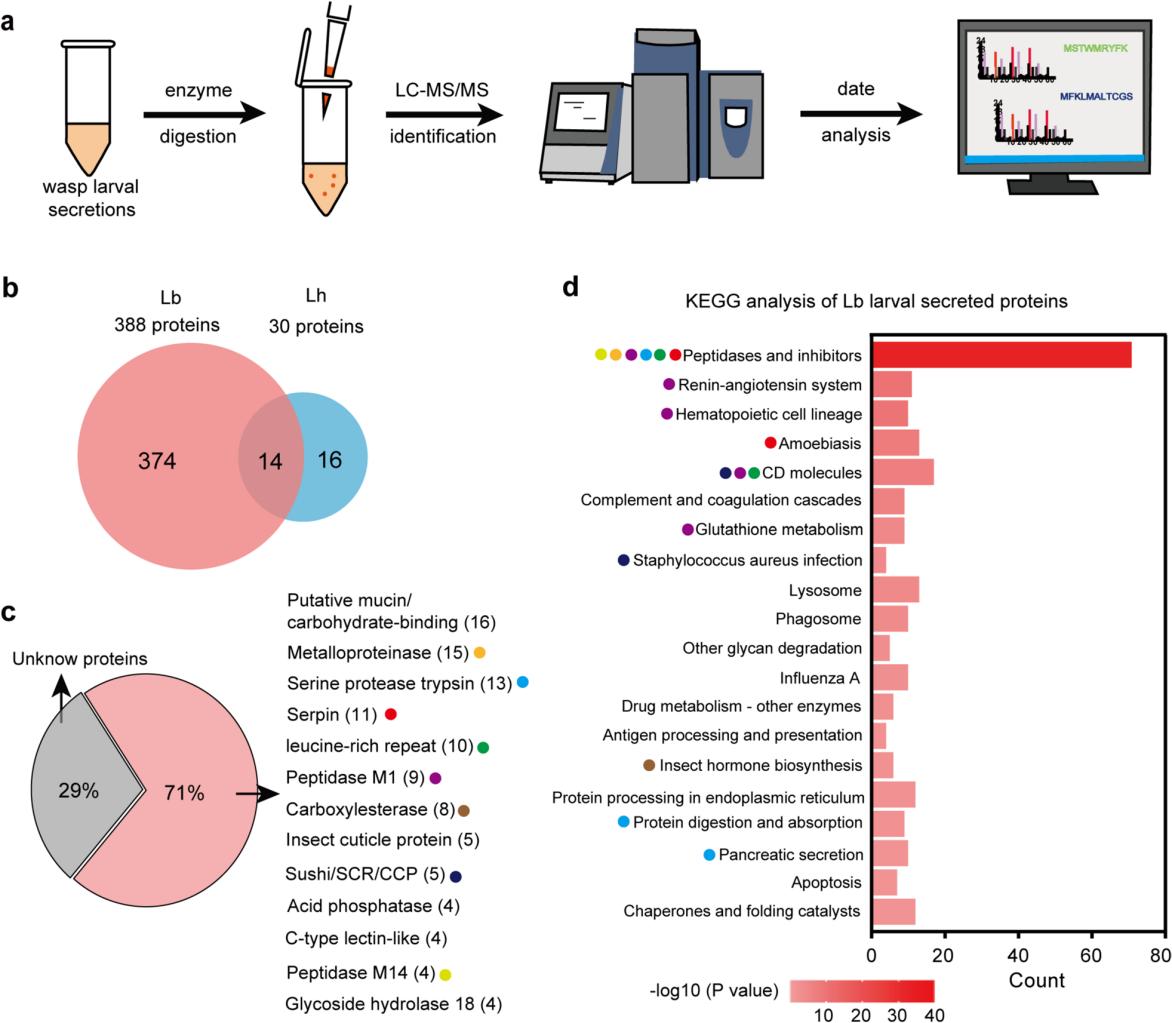
Identification of Lb and Lh larval secretions by LC-LS/MS. **a** Flow chart for LC-LS/MS identification of wasp larval secretions. **b** Venn diagram of secreted proteins of Lb and Lh larvae. The unoverlapped portion represents species specific proteins. **c** Proteins identified in Lb not Lh larval secretions. **d** KEGG pathway enrichment analysis for the Lb-specific larval secretory proteins. Representative top 20 significantly enriched KEGG pathways were shown

What component of wasp secretions plays an essential role in manipulating host immune responses? To obtain a complete catalogue, we sampled larval secretions of the two parasitic wasps and further sequenced the proteome through LC‒MS/MS analysis (Fig. 5a). In combination with the transcriptome of Lb and Lh wasps, 388 secretory proteins were identified as reliable Lb secretions in a total of 7540 larva-expressed genes; meanwhile, 30 secretory proteins were identified for Lh in 7474 larva-expressed genes (Fig. 5b) (Online Resource 2, Table S1 and S2). The number of Lb secretory proteins was much higher than that of Lh, which may explain our previous findings that Lb larval secretions impair host immune responses while Lh larval secretions do not. We then focused on the 374 Lb-specific secretory proteins, among which 266 (71%) were annotated proteins and the remaining 108 (29%) were found to be unknown proteins. Within the annotated proteins, there were several main categories, including putative mucin/carbohydrate-binding proteins, metalloproteases, serine proteases, serpins, leucine-rich repeat proteins, M1 peptidases, carboxylesterases, and others (Fig. 5c). Moreover, we performed KEGG analysis of the 374 Lb-specific secretory proteins and identified 39 significantly enriched KEGG pathways (Online Resource 2, Table S3). Among them, the KEGG pathway of peptidases and inhibitors was notably enriched, including serpins, metalloproteases, serine proteases, and leucine-rich repeat proteins (Fig. 5c and d). Interestingly, peptidases and inhibitors have been reported to be involved in regulating insect immunity [45–47].

### Serpins in Lb larval secretions inhibit host melanization

Upon infection, a series of serine proteases are sequentially activated, leading to the activation of PPO activating proteinase (PAP). Activated PAP further converts PPO (inactive phenoloxidase) to PO (active phenoloxidase),which triggers the melanization response [42, 48, 49]. Serine protease inhibitors (serpins) are known to target the PO cascade and usually inhibit PO activity [50, 51]. For instance, a Lb serpin gene (*LbSPNy*) is highly expressed in the venom gland and has the function of inhibiting host PO activity [52].

**Fig. 6:**
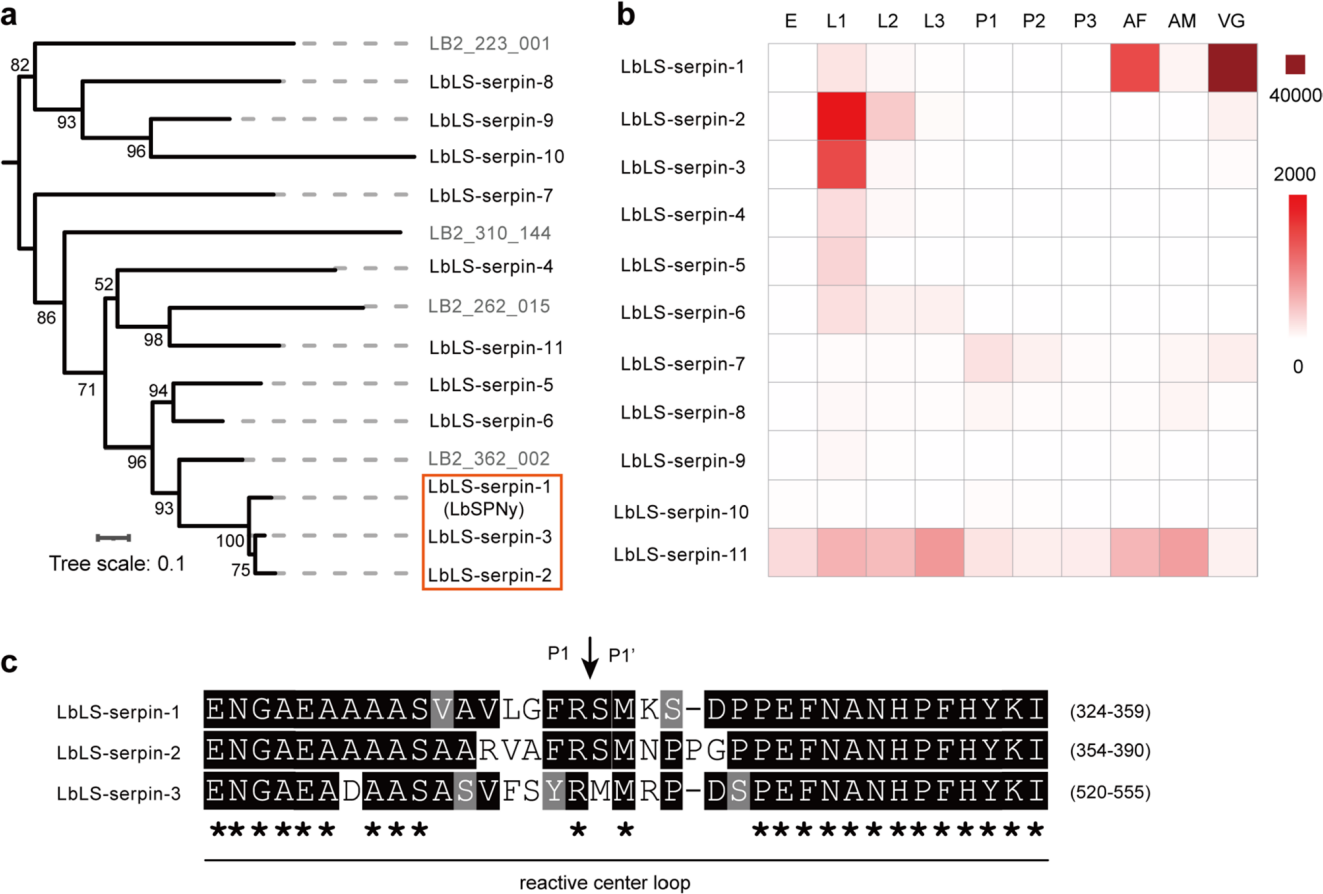
Serpins in Lb larval secretions. **a** Phylogenetic tree of all serpin genes in the Lb genome. The serpin genes with black font were present in the larval secretions, while serspin genes with grey font were not found in larval secretions. The serpin sequences used for this tree are shown in Online Resource 2, Table S4. The genes with high sequence similarity to *LbLS-serpin-1* (*LbSPNy*) were highlighted in the red box. **b** Expression heatmap of serpin genes present in Lb larval secretions. E, eggs; L1, days 1–3 larvae; L2, days 4–6 larvae; L3, days 7–9 larvae; P1, days 1–3 pupae; P2, days 4–7 pupae; P3, days 8–10 pupae; AF, female adults; AM, male adults; VG, venom glands. **c** Multiple alignments of reactive central loop (RCL) of serpins. The predicted P1-P1′cleavage site was indicated with an inverted black arrow. Residues identical or similar to those in LbLS-serpin-1 sequence were highlighted in black and grey, respectively. ‘‘*’’ marks positions with identical residues in above sequences

We further analysed the potential serpins in the Lb genome. There were 15 serpin genes in total, and 11 of which were expressed in Lb larvae and detected in Lb larval secretions. They were termed as *LbLS-serpin-1* to *LbLS-serpin-11* (Online Resource 2, Table S4). Notably, *LbLS-serpin-1* was a previously reported *LbSPNy* gene [52]. The phylogenetic analysis revealed that *LbLS-serpin-1*, *LbLS-serpin-2*, and *LbLS-serpin-3* clustered into the same group with more than 85% sequence identity at the nucleotide level (Fig. 6a) (Online Resource 1, Fig. S2). In addition, *LbLS-serpin-2* and *LbLS-serpin-3* were highly expressed at the Lb larval stage (Fig. 6b). These features indicate that *LbLS-serpin-2* and *LbLS-serpin-3* might play dominant roles in inhibiting host PO activity and the subsequent melanization response.

The serpin reactive center loop (RCL) is responsible for interaction with the active site of target serine proteases, and the P1-P1’ site in RCL determines the specificity [50, 53]. We further compared the RCL sequences of LbLS-serpin-1, LbLS-serpin-2, and LbLS-serpin-3. The results showed that the RCL sequences of LbLS-serpin-2 and LbLS-serpin-3 were conserved with that of LbLS-serpin-1 (Fig. 6c). Moreover, the P1-P1’ residues were also much similar (Fig. 6c), indicating that these serpin proteins might target the same serine proteases in the PO cascade.

### Metalloproteases in Lb larval secretions inhibit host cell adhesion

**Fig. 7:**
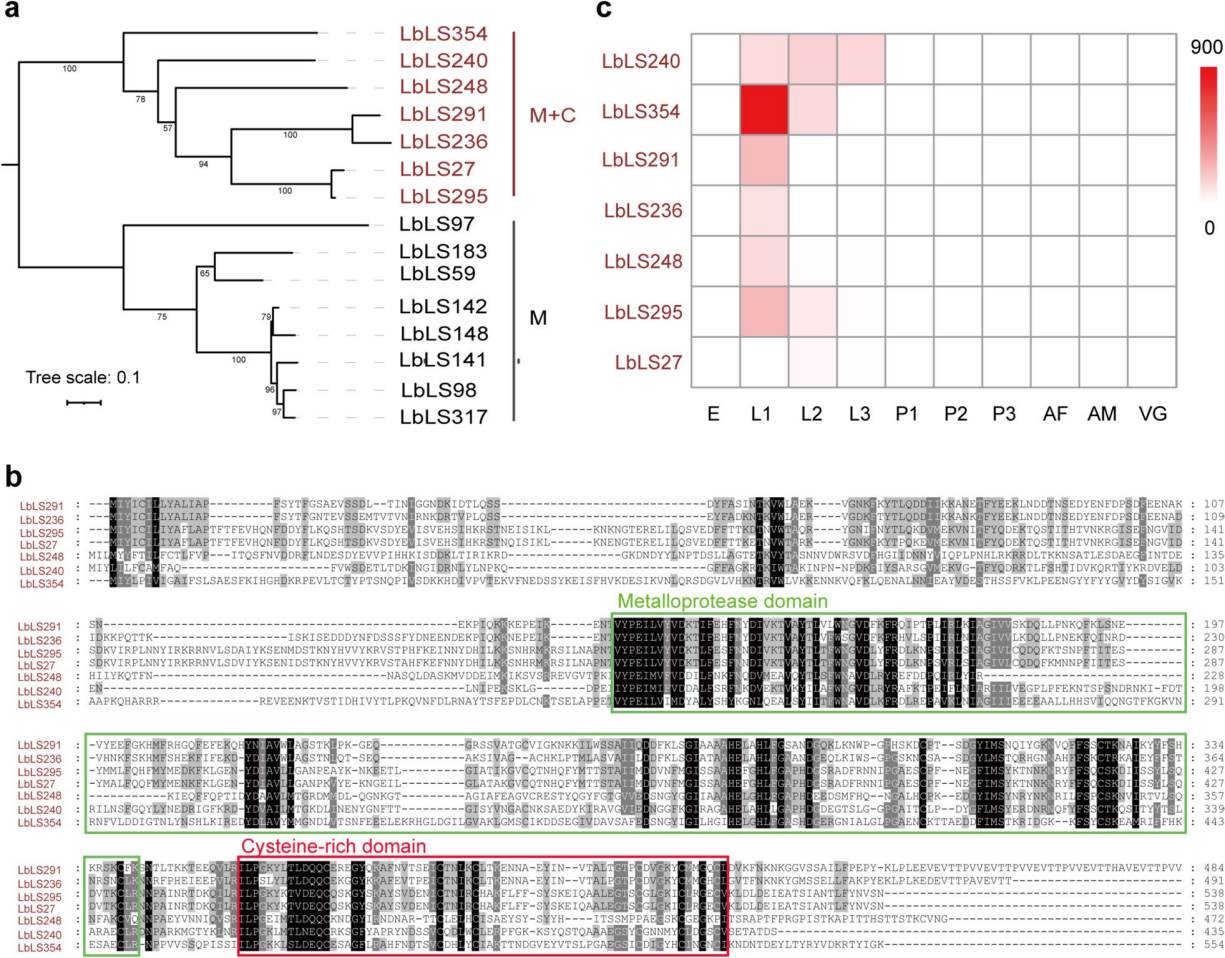
Metalloproteases in Lb larval secretions. **a** Phylogenetic tree of metalloproteinase in Lb larval secretions. The sequences used for this tree are shown in Online Resource 2, Table S5. The genes with red font contained metalloproteinase (M) and cysteine-rich (C) domains and the genes with black font only contained metalloproteinase domains. **b** Multiple alignments of metalloproteinase proteins in Lb larval secretions containing metalloproteinase and cysteine rich domains. Residues identical or similar to those sequences were highlighted in black and grey, respectively. The metalloproteinase domain was marked by a green box and the cysteine-rich domain was marked by a red box. **c** Expression profiles of metalloproteinase genes in Lb larval secretions containing metalloproteinase and cysteine rich domains at different developmental stages

The cell adhesion process depends on integrins, which mediate the attachment of cells to the extracellular matrix (ECM) [54, 55]. It has been reported that snakes have evolved many disintegrin venom proteins to destroy the cell adhesion of their prey [56, 57]. These specialized venom proteins belong to the metalloproteinase superfamily, normally containing one metalloproteinase domain and one cysteine-rich domain [58–60]. Strikingly, a total of 15 metalloproteinase proteins were found in Lb secretory proteins (Fig. 5c) (Online Resource 2, Table S5). These 15 metalloproteinase were divided into two categories, one group had 8 genes that only contained metalloproteinases domain, and the other group had 7 genes that contained both metalloproteinase domain and cysteine-rich domain (Fig. 7a and b). Moreover, the 7 metalloproteinase genes with metalloproteinase and cysteine-rich domains were highly expressed at the Lb larval stage (Fig. 7c). These results suggest that parasitoid Lb secretes a group of metalloproteinases (similar to snake disintegrin venom protein) that potentially impair the adhesion of host immune cells and the subsequent encapsulation response.

## Discussion

Parasitoid wasps comprise the most abundant group of insects and serve as crucial agents against pest populations in natural agricultural ecosystems. In order to gain successful parasitism, the parasitoids have evolved numerous strategies to circumvent the host immune defenses by using of a series of effectors, including venom, PDVs and teratocytes [2, 16–18]. Previous studies have made considerable achievements in understanding how venom, PDVs, and teratocytes regulate host immunity [16, 17, 26, 27]. In this study, we examined two species of *Leptopilina* endoparasitoid wasps (Lb and Lh) with contrasting strategies to circumvent host immunity, and found a new type of parasitoid effectors, the larval secretions.

Based on LC‒MS/MS analysis, 388 proteins were identified as reliable Lb (evasion strategy) larval secreted proteins, while only 30 larval secreted proteins were detected for Lh (suppression strategy). The concentration and number of larval secreted proteins of Lh were significantly lower than those of Lb. These findings may explain why Lb larval secretions can suppress host immune responses, while Lh larval secretions do not. Specifically, there were 11 serpins in Lb larval secretions, but not in Lh. The parasitoid serpins are well-known to inhibit host melanization. For example, a serpin from venom of the endoparasitoid wasp *P. puparum* is capable of inhibiting PO activity by forming complexes with the hemolymph proteinase 8 and prophenoloxidase-activating proteinase 1 of *Pieris rapae* host; serpin6 from *Cotesia vestalis* teratocytes can inhibit the activation of PPO and melanization of its host, *Plutella xylostella* [61, 62]. In this study, we found that the serpins in Lb larval secretions had the conserved RCL domain and P1-P1’ site with the previously described wasp serpins [52], indicating that they are responsible for regulating the host melanization response by inhibiting PO activity. Serpins have been reported to affect some step(s) of the cascade leading to PO activation by forming complexes with their serine protease targets [51, 63]. The serpins in Lb larval secretions have an Arg (R) at the predicted P1 site, suggesting that they may inhibit a group of serine proteases with trypsin-like specificity [64]. However, the exact target proteases of serpins in Lb larval secretions need further investigation.

Parasitoid wasps also have the ability to impair the adhesion of host hemocytes to overcome the host encapsulation response. For instance, it has been reported that the venom of *Asobara japonica* significantly disrupted the adhesion behavior of *D. melanogaster* hemocytes [65]. The venom fluid of *N. vitripennis* reduced the spreading and adhesion activity of host hemocytes [66]. However, the molecules used by parasitoid wasps to break host hemocyte adhesion are not well-documented. So far, the only known factor of parasitoid wasps that regulates host hemocyte adhesion is the RhoGAP protein. The RhoGAP venom protein has the ability to destabilize the lamellocyte cytoskeleton, leading to changes in lamellocyte morphology and ultimately preventing the host from encapsulating the wasp eggs [67, 68]. However, we did not find RhoGAP proteins in the secretions of Lb larvae. Instead, we identified 7 metalloproteinases in Lb larval secretions that contain both a cysteine-rich domain and a metalloprotease domain. These metalloproteinases shared the similar domains with the disintegrins of snake venom, which are reported to interfere with cell–cell and cell-ECM interactions by blocking the function of integrin receptors [56–59]. As such, we speculate that these disintegrin-like metalloproteases in Lb larva secretory proteins might potentially play a role in inhibiting the adhesion of host hemocytes and thus impede the resulting encapsulation response. Further studies are needed to confirm whether these disintegrin-like metalloproteases in Lb larval secretions impair the adhesion of host hemocytes by destroying the integrin-mediated cell–matrix and cell‒cell interactions [57, 69]. Moreover, EMS16, Rhodocetin, and VP12 are three toxins found in snake venom that affect the cell adhesion of the prey [70, 71]. These proteins belong to the C-type lectin family. In the Lb larval secretions, we also identified 4 C-type lectin proteins that were not detected in Lh larval secretions. Among them, two were found to be highly expressed at the larval stage (Online Resource 1, Fig. S3). These results suggest that there may be other secretory proteins ruining host hemocyte adhesion besides the metalloproteinases, when the hosts are infected by parasitoid wasps.

In conclusion, our results have revealed that larval secretions serve as new effectors of parasitoids. The components of larval secretions were uncovered and the potential secreted proteins were discovered with the function of inhibiting host melanization and encapsulation responses. However, the detailed mechanisms are necessary to be explored in the future. Nevertheless, our findings contribute to the understanding of the strategies employed by parasitoids to regulate host immune responses and the coevolutionary arms race between parasitoids and their hosts.

## Materials and Methods

### Insects

*D. melanogaster* (hosts) were maintained on cornmeal/yeast/sugar *Drosophila* medium (the recipe can be found on Bloomington Drosophila Stock Center, https://bdsc.indiana.edu/information/recipes/bloomfood.html) at 25 °C, 50% humidity, and a 16 h: 8 h light: dark cycle. *Canton-S* (BL64349), *HOP*^*Tum−1*^ (BL8492), and *Hml-GAL4* > *UAS-GFP* (*Hml* > *GFP*, BL30140) were acquired from the Bloomington Drosophila Stock Center. *MSNF9MO-mCher*ry (msnCherry) was provided by Robert Schulz (University of Notre Dame, USA). *Canton-S* was used as a wild-type *D. melanogaster* strain.

The parasitoids *L. boulardi* (Lb) and *L. heterotoma* (Lh) were maintained in our lab since 2016 [22]. Parasitoid wasps were maintained on *Canton-S* in our lab under the following conditions. Briefly, 50 mated *Drosophila* females were placed into a fly bottle (top: 3.5 cm diameter; bottom: 5.7 cm length and 5.7 cm width; height: 10.3 cm; volume: 200 cm^3^) containing cornmeal fly food. The flies were allowed to lay eggs for 2 h. After the *Drosophila* eggs hatched to the 2nd instar (approximately 48 h later), 10 mated parasitoid females were added to each bottle and allowed to parasitize the hosts for 6 h. The parasitized larvae were maintained at 25 °C until adult wasps emerged. The newly emerged male and female wasps were collected and allowed to mate in vials with apple juice agar medium (27 g agar, 33 g brown sugar and 330 ml pure apple juice in 1000 ml diluted water) for further use.

### Wasp parasitism assay

Flies were allowed to lay eggs on medium at 25 °C for 2 h to maintain about 300–500 eggs per bottle. After the *Drosophila* eggs hatched to the 2nd instar, one hundred host larvae were carefully transferred onto fresh food dishes. These transferred host larvae were then combined with 20 mated Lb females or 10 mated Lh females of 3 days post-emergence for 1 h. The oviposition rate and wasp emergence rate were calculated using the following formulas:

$$\mathrm{Oviposition}\;\mathrm{rate}=(\mathrm{number}\;\mathrm{of}\;\mathrm{hosts}\;\mathrm{containing}\;\mathrm{wasp}\;\mathrm{eggs}/\mathrm{number}\;\mathrm{of}\;\mathrm{total}\;\mathrm{hosts})\times100\%$$

$$\mathrm{Wasp}\;\mathrm{emergence}\;\mathrm{rate}=(\mathrm{number}\;\mathrm{of}\;\mathrm{emerged}\;\mathrm{wasps}/\mathrm{number}\;\mathrm{of}\;\mathrm{total}\;\mathrm{hosts})\times100\%$$

To observe the hatching time of wasp eggs, the parasitized hosts were dissected every 6 h and the number of hatched eggs was counted. The hatching rate was calculated using the following formula:

$$\mathrm{Hatching}\;\mathrm{rate}=({\mathrm{number}\;\mathrm{of}\;\mathrm{hosts}\;\mathrm{containing}\;\mathrm{hatched}\;\mathrm{larvae}}/\mathrm{number}\;\mathrm{of}\;\mathrm{total}\;\mathrm{hosts})\times100\%$$

### Quantification of host hemocytes

At 24 h, 48 h, and 72 h post parasitization, host larvae were rinsed thrice with PBS and dried with filter paper. Next, the hemolymph of 15 host individuals was diluted in 30 μL of PBS, and 8 μL of the mixture was dropped on the hematocytometer. The number of host hemocytes was counted using a hematocytometer (Watson) under a Zeiss LSM 800 confocal microscope. Circulating hemocytes were detected with fluorescent GFP (*Hml* > *GFP*) and lamellocytes were detected with fluorescent Red (*msnCherry*), respectively.

### Confocal microscopy

To collect the images of host hemocytes, both parasitized and nonparasitized *Hml* > *GFP*; *msnCherry* larvae were washed three times in PBS at 24 h, 48 h and 72 h post parasitization. Hemocytes were then bled from three individuals per group into 30 μl of PBS and allowed to adhere to a glass surface of a microscope slide for 30 min. Next, the hemocytes were fixed with 4% paraformaldehyde for 10 min. The fluorescence images were captured on a Zeiss LSM 800 confocal microscope.

To collect the images of wasp eggs, the eggs were dissected from *Hml* > *GFP*; *msnCherry* host larvae in PBS at 24 h post parasitization, and then fixed with a 4% paraformaldehyde solution for 10 min. The fluorescence images were captured on a Zeiss LSM 800 confocal microscope.

### Wasp larval secretions collection

Parasitized 3rd instar host larvae were collected and washed thrice in PBS. The epidermis of the host larvae was carefully torn open under a Leica M125 stereomicroscope, and wasp larvae were washed eight times in Schneider's medium (Thermo Fisher Scientific, Cat# 21720001). The wasp larvae were then allowed to grow in a certain amount of Schneider's medium containing 1% Penicillin–Streptomycin at 25 °C, approximately 2 μl medium for each wasp larva. After 24 h, the wasp larval secretions were collected and stored at -80 °C for further use.

### PO activity measurement

PO activity was determined according to previous studies with some modifications [72, 73]. Briefly, about 10 μl of host hemolymph from 3rd instar larvae were collected and immediately suspended in 10 µl of Schneider's medium, 10 µl of PTU (0.01% 1-phenyl-2-thiourea), 10 µl of collected Lb larval secretions (1:1), 10 µl of Lb larval secretions (1:5), and 10 µl of Lh larval secretions (1:1). The different ratios represent the dilutions of secretions collected from wasps as previously described. The mixture was then centrifuged at 8,000 g for 5 min, after which the supernatant was transferred into a new tube. After 30 min incubation at room temperature, during which PPO is converted into PO, 200 µl substrate (50 ml of 5 mM L-DOPA prepared in PBS) was added. The plate was further incubated for 1 h at room temperature, and the level of PO activity was estimated spectrophotometrically by measuring the optical density (OD) at 520 nm.

### Hemocyte adhesion ability assay

*HOP*^*Tum−1*^ larvae contain both plasmatocytes and lamellocytes, which mimic the hemocytes in wasp infected hosts. The *HOP*^*Tum−1*^ 3rd instar larvae were washed thrice in PBS, after which hemocytes from three individuals were bled onto a glass slide containing different solutions including 30 μl of Schneider's medium, 30 µl of collected Lb larval secretions (1:1), 30 µl of Lb larval secretions (1:5), and 30 µl of Lh larval secretions (1:1). The different ratios represent the dilutions of secretions collected from wasps as previously described. The hemocytes were then allowed to adhere to the glass surface for 30 min, followed by fixing with 4% paraformaldehyde for 10 min. The fixed samples were incubated with 1% bovine serum albumin (BSA) for 45 min, followed by staining with 1:1000 phalloidini Fluor 488 (Invitrogen, Cat# A12379) diluted in 1% BSA for 1 h to visualize the F-actin of hemocytes. The stained hemocytes were then mounted in ProLong Gold Antifade Mountant with DAPI (Invitrogen, Cat# P36941) and photographed using a Zeiss LSM 800 confocal microscope. Plasmatocytes are small spherical cells and lamellocytes are large discoid cells. In the cell adherence ability analysis, the cells with a fluorescently stained area larger than 400 mm^2^ were considered as lamellocytes.

### Wasp larval secretions identification

To identify the proteins in wasp larval secretions, approximately 1000 wasp larvae were cultured and the protein concentration was determined using the BCA protein detection kit (Invitrogen, #23,225). The proteins were then subjected to proteomic identification via LC‒MS/MS analysis. Firstly, the secreted proteins were digested into short peptides by trypsin. Then, 5 μg of the enzymolytic products were taken for LC‒MS/MS analysis on a Q Exactive mass spectrometer (Thermo Fisher Scientific), which was coupled to an Easy nLC HPLC liquid system (Thermo Fisher Scientific). The 5 μg peptide mixture was loaded onto a reverse-phase trap column (Thermo Fisher Scientific, Acclaim PepMap 100, 100 μm × 2 cm, nanoViper C18), which was connected to the C18 reversed-phase analytical column (Thermo Fisher Scientific Easy Column, 10 cm long, 75 μm inner diameter, 3 μm resin). The mixture was separated with a linear gradient of buffer B (84% acetonitrile and 0.1% formic acid) at a flow rate of 300 nl/min, with the column being initially equilibrated in buffer A (0.1% formic acid). The separated peptides were ionized, and the full MS spectrum (ranging from m/z 300 to 1800) was acquired by precursor ion scan using the Orbitrap analyser, with a resolution of *r* = 70,000 at m/z 200. This was followed by 20 MS/MS events in the Orbitrap analyser, with a resolution of *r* = 17,500 at m/z 200. The MS raw files were converted to mgf files and queried against Lb and Lh reference genomes utilizing Mascot 2.2 [74]. The MS/MS tolerance was set at 20 ppm with trypsin being designated as the cleavage enzyme, allowing no more than two missed cleavages. Carbamidomethylation of cysteine was selected as a fixed modification, while oxidation of methionine was chosen as a variable modification.

The genes of wasp larval secretions were determined based on a combination of transcriptomic and proteomic evidence. The transcriptomes from different developmental stages and reference genomes of Lb and Lh were acquired from our previously published data [22, 75]. Transcriptome expression analysis was performed independently and mapped to the reference genome utilizing HISAT2 v2.0.5, with default parameters [76]. Genes with TPM values greater than 5 in the wasp larval transcriptome were designated as larva-expressed genes. These gene sets were then used to perform alignment with the proteomic data, and those that exhibited a completely aligned with at least three proteomic peptides were identified as wasp larval secretion genes (Online Resource 2, Tables S1 and S2).

### Annotation and sequence analysis

The genes of wasp larval secretions were annotated with functions using EggNOG-Mapper v2 against eggNOG databases and a local InterProScan search (v5.38–76.0) for domains [77, 78]. Multiple sequence alignment was performed with the ClustalX version 1.83 software. To construct the phylogenetic tree, coding sequences were aligned using MAFFT (v7.487) [79] and trimmed using trimAl (v1.4 rev15) [80]. A maximum likelihood phylogeny was then computed with IQ-TREE v2.1.3 with 1,000 bootstraps [81], and the best model for each partition was assessed with ModelFinder (option -m MFP) [82].

### Statistics

Statistical analyses were performed in GraphPad Prism version 8.0 (GraphPad Software) and SPSS 26 (IBM). The Shapiro‒Wilk test was used to test the normal distribution of the data and the Bartlett chi square test was used to test the homogeneity of variance of the data. Statistical significance was determined using two-tailed unpaired Student’s t test (parametric) and Mann‒Whitney U test (nonparametric) when comparing two treatments. For comparisons of multiple group experiments, one-way of variance (ANOVA) with Sidak's multiple comparisons test was used for normally distributed data. The Kruskal‒Wallis test was used for multiple groups of experiments requiring nonparametric statistical tests. Details of the statistical analysis were provided in the figure legends, including how significance was defined and the statistical methods used. Data represented the mean ± standard error of the mean (SEM). Different letters in Figs. 2b, c, 3b, 4b and c indicate statistically significant differences (*p* < 0.05). For other tests, significance values are indicated as ** p < 0.01; *** p < 0.001.

## Supplementary Information

**Additional file 1:** **Fig. S1.** The protein concentration of Lb and Lh larval secretions. **Fig. S2.** Sequence alignment between *LbLS-serpin-1*, *LbLS-serpin-2 *and *LbLS-serpin-3 *genes. **Fig. S3.** Expression profiles of C-type lectin-like genes in Lb larval secretions.

**Additional file 2:** **Table S1.** Lb larval secretions. **Table S2.** Lh larval secretions. **Table S3.** Enriched KEGG pathways of Lb-specific secretory proteins. **Table S4.** Serpins in Lb larval secretions. **Table S5.** Metallopeptidase in Lb larval secretions.

## Data Availability

The MS proteome data were deposited to the ProteomeXchange Consortium (http://proteomecentral.proteomexchange.org) through the PRIDE partner repository (https://www.ebi.ac.uk/pride/) with the dataset identifier PXD043741. Other datasets generated during and/or analysed during the current study are available from the corresponding author on reasonable request.
