# Supplementary material for: Larval secretions of parasitoid wasps are new effectors that impair host immune defences
Source: Crop Health. 2023 Nov 15;1(1):11. doi: 10.1007/s44297-023-00011-y (PMC12825982; doi:10.1007/s44297-023-00011-y)
Supplement: Supplementary file 1 — Additional file 1: Fig. S1. The protein concentration of Lb and Lh larval secretions. Fig. S2. Sequence alignment between LbLS-serpin-1, LbLS-serpin-2 and LbLS-serpin-3 genes. Fig. S3. Expression profiles of C-type lectin-like genes in Lb larval secretions. [file 44297_2023_11_MOESM1_ESM.pdf]

Supplementary information (Online Resource 1) for

Larval secretions of parasitoid wasps are new effectors that impair host immune defences

Lan Pang[1,2,3], Zhi Dong[1,2], Zhiguo Liu[1,2], Feng Ting[1,2], Wenqi Shi[1,2], Yueqi Lu[1,2], Yifeng Sheng[1,2], Jiani Chen[1,2,3], Xueying Guan[3], Xuexin Chen[3,4], Jianhua Huang[1,2*]

[1] Institute of Insect Sciences, Ministry of Agriculture Key Lab of Molecular Biology of Crop Pathogens and Insect Pests, Zhejiang University, Hangzhou, China
[2] Key Laboratory of Biology of Crop pathogens and Insects of Zhejiang Province, Zhejiang University, Hangzhou, China
[3] Zhejiang Provincial Key Laboratory of Crop Genetic Resources, Institute of Crop Science, Plant Precision Breeding Academy, College of Agriculture and Biotechnology, Zhejiang University, Hangzhou, China
[4] Guangdong Laboratory for Lingnan Modern Agriculture, Guangzhou, China

*Corresponding author. Email: jhhuang@zju.edu.cn (J.H.)

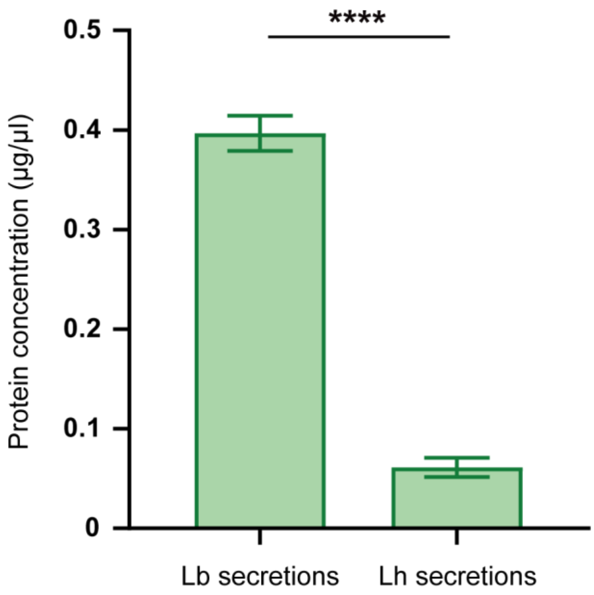

Fig. S1 The protein concentration of Lb and Lh larval secretions

Three independent biological replicates were performed. Data represent the mean ±SEM.

Significance was analyzed by two-tailed unpaired Student's t test (****p < 0.0001).

```
LbLS-serpin-2 --------------------------------ATGATGTTCACACATTTACTGTCGTTTTATCTAATAGGTACTTGTAGTATTTATGCAGTAATG :   63
LbLS-serpin-3 TTATTAGAACAGTTGGTGAACATGATGGTATTGCATTGTTTGCTGATTTACTGTCGTTTTTTCTAATAGGTTCTTGTAGTATTTATACAGTAATT :  570
LbLS-serpin-1 --------------------------------ATGATGTTCACACATTTACTGTCGTTTTTTCTAATAGGTACTTGTAGTATTTATGCAGTAATT :   63

LbLS-serpin-2 GCTTATGGTAACGTAAATTACTTTAGTGAAGAATTGGTACAACGACTCGACTTTTATCAAGCCTTGGAAAAATTCAATAACGACTTATACAAGAA :  158
LbLS-serpin-3 GCTTATGGTAACGTAAATTACGATAATGCAGAACC---------ACTCAACTTTTATGATATCTTGGAAAACTTCAATAACAACTTATACAAGAA :  656
LbLS-serpin-1 GCTTATGGTAACGTAAATTACTTTAGTGAAGAATTGGTACAACCACTCGACTTTCATCAAGCCTTGGAAAAATTCAATAACGACTTATACAAGAA :  158

LbLS-serpin-2 TGCACCGTCAAAAGGATCCATACAAAATGTTTTATTATCTCCTTTGAGTATAAATATACTCCTAGCTATACTTGCCGTTGGAGCTGGAGGAAGAA :  253
LbLS-serpin-3 TGCAGCGTCAAAAGGATCCATACAAAATGTTTTATTATCTCCTTTGAGTATAAATATACTCTTAGCTATACTTGCCGTTGGAGCTGGAGGAAGAA :  751
LbLS-serpin-1 TGCAGCGTCAAATGGATCCATACAAAATGTTTTATTATCTCCTTTGAGTATAAATATACTCTTAGCTATACTTGCCGTTGGAGCTGCAGGAAGAA :  253

LbLS-serpin-2 CTAGATCTCAAATAATCACAGCTCTCAATCAGCCAGTCCAGTCGGGCGCACAAATTTTAAATAATTATAAGTTGATTATGGAAAATTGGATGAAT :  348
LbLS-serpin-3 CTAGATCTCAAATAGTCACAGCTATCAATCAGCCATCCCAGTCGGGCGCACAAATTTTAAATAATTATAAGTTGATTATGGAAAACTGGATGAAT :  846
LbLS-serpin-1 CTAGATCTGAAATAGTCACAGGTATCAATCAGCCATTACAGTCGGGCGCACAAATTTTAAATAACTATAAGTTGATGGTGGAAAACTTGATGAAT :  348

LbLS-serpin-2 GTCACAGGTGCAGAATTAGAAATTTCTAACGCAGTATTCTTCAGTGATTGTCTAAAACTTAAGAGAGAATTTTATCACGAATTATTATTTTACTT :  443
LbLS-serpin-3 GTCACAGATGCAGAATTACAAATTTCTAACGCAATATTCGTCAGTCATAGTCTAAGACTTAAGGAATCATTTAGATACGAATTATTTAATTACTT :  941
LbLS-serpin-1 GTCACAGATGTAGAATTACAAATGTCTAACGCAATATTCGTCGATCATAGTATAAGACTTAAGAAATCATTTCAAGACGAATTATTTAATTACTT :  443

LbLS-serpin-2 TAAAGCACATGCATTTTTAGCGAATTTTGGAACCCCAACAGCAACAGCAAATAAGATTAATAACTGGATTTCGACACAAACAAATAACAAAATCA :  538
LbLS-serpin-3 TAAAGCACATGAATTTTTAGCGAATTTTGGAACTCCAACAGCAACAGCAAATGCGATTAATGGATGGATTTCGAGACAAACAAATAACAAAATCA : 1036
LbLS-serpin-1 TAAAGCACATGAATTTTCAGTGAATTTTAAAACTCCAATACCAACAGTAGATAAGATTAATGGAAAGATTTCGGAACAAACAAATAACAAAATCA :  538

LbLS-serpin-2 ATAATGTATTATCACCAAATGATATAGATGTAACATTTACTAAAATGGTCCTCACTAATGTCATTCACTTTAAAGGTGAATGGAAGTATAAGTTC :  633
LbLS-serpin-3 ACAATATATTATCACCAAATGATATAGATGTAGCATCTACTAAAATGGTCCTCACTAATGCCATTCACTTTAAAGGTGAATGGAAGAATAAGTTC : 1131
LbLS-serpin-1 ACAATATGTTATCAACAAAGGATATAGATGAACGAAGTACTAAAGTAGTCATCACTAATGCCATTTACTTTAAAGGTGAATGGAAGTATAAGTTC :  633

LbLS-serpin-2 TCCGATGTTACTAATTTGTTCTTTTATGACTATCATGGTCAAACTAAAATCGTACCTACTATGACTCAAACAGCTCAATATAGAGTGGCACATGT :  728
LbLS-serpin-3 AACGTTGTTACTAATTTGTTCTTTTATGGCTATAATCATCAAACTACTAACGTACCTACTATGATTCAAATAGCTGAATATAGATTTGTACATGT : 1226
LbLS-serpin-1 GCCAATGTTACTAATTTGGTCTTTCATGACTATCATGGTCAAACTAAAATCGTACCTACTATGACTAAAACAGGTCCATATAGAGTGTCATTTGG :  728

LbLS-serpin-2 ---TCCTGCAATAAAAGCTAATATCATAGAATTGCCATATAAGGGTGATGAACTTAGTATGCTTATCGTACTTCCTGATGAAATGTATGGTCTGG :  820
LbLS-serpin-3 ---TCCTGCAATAAAAGCTAAATTCATAGAATTGCCATATAAGGGTGACGAACTTAGTATGCTTATCGTGCTTCCTGATGAAATGTATGGTTTGG : 1318
LbLS-serpin-1 AGATACAGCAATAAAGGCTCGAATGATAGAATTGCCATATAAGGGTGACGAACTTAGTATGCTTATCGTGATTCCTTATGAAATGAATGGTCTGG :  823

LbLS-serpin-2 ACGATGTTGAAAGTAGTTTAGAGAGAGTAAACCTTAGAAACCTCAGAAATTCTCTATCGCTTATTACAGTTAAATTGAATTTACCTAAATTTCGG :  915
LbLS-serpin-3 ACGATGTTGAAAGTATTTTAGAGAGAGTAAACCTTAGAGTCCTCAGAAAATCTCTATCGCTTATTAAAGTTAAATTGAATTTGCCTAAATTTCGG : 1413
LbLS-serpin-1 ACGATGTTGAAAGTAGTTTAGAGAGAGTAAACCTTAGAAACCACATAAAATCTCTAATGCCTTTTGAAGTTAAATTGAATTTGCCTAAATTTCGG :  918

LbLS-serpin-2 GTTGAAGCTACCACTAACTTGAATGATGCTTTATATAAGATGGGTATAAATGATTTGTTTACGGATTTCGCAAACTTCACTCGCATTACAGATGA : 1010
LbLS-serpin-3 GTTGAAGCTACCACTGACTTGTATGATGCTTTACATAAGATGGGTATAAATGATTTGTTTATGGATGTCGCAAACTTCACTCGCATTACAGATGA : 1508
LbLS-serpin-1 GTTGAAGCTACCACTGACTTGTATGATGCTTTAAATAAGATGGGTATAAATGAAGCGTTTACAGATATCGCAAACTTCAGTCGCATTACAGATGG : 1013

LbLS-serpin-2 CATAATTGGTGGGTGTAAAATTATCCATAAAACTTTTATTGAAGTCAACGAGAATGGTGCTGAAGCTGCTGCAGCTTCAGCTGCACGTGTCGCCT : 1105
LbLS-serpin-3 CATAATTGGTGGGTGTAAAATTATGCATAAAACTTTTATTGAAGTCAATGAGAATGGTGCTGAAGCTGATGCAGCTTCAGCTTCAGTTTTCAGCT : 1603
LbLS-serpin-1 AAATCTTTCTGTGAGTAAAATGATGCATAAAACTTTAATTGAAGTCAATGAGAATGGTGCTGAAGCTGCTGCAGCTTCAGTTGCAGTTTTAGGGT : 1108

LbLS-serpin-2 TTAGATCAATGAATCCCCCTGGTCCTCCAGAATTTAATGCTAATCATCCGTTTCATTATAAAATTATTAAATCAGTTGATGAAAATAATGATGTT : 1200
LbLS-serpin-3 ACAGAATGATGAGACCC---GATTCTCCTGAATTTAATGCTAATCATCCGTTTCATTATAAAATTATTAAATCAATTGATGAAAATAATCATGTT : 1695
LbLS-serpin-1 TCAGATCGATGAAATCC---GATCCTCCTGAATTTAATGCTAATCATCCGTTTCATTATAAAATTATTAAATCAATTGATAAAAATAATCATGTT : 1200

LbLS-serpin-2 GTATTGTTTGCTGGTAACGTCAAACGTATAGTGTAA : 1236
LbLS-serpin-3 GTATTGTTTGCTGGTAACGTCAAACATATACAGTAA : 1731
LbLS-serpin-1 GTATTGTTTGCTGGTAACGTCAAACATATACAGTAA : 1236
```

Fig. S2 Sequence alignment between *LbLS-serpin-1*, *LbLS-serpin-2* and *LbLS-serpin-3* genes

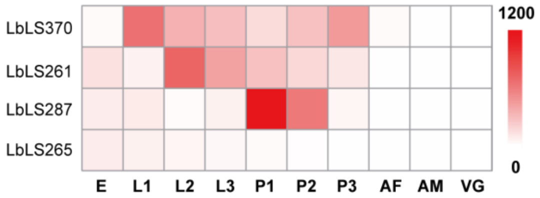

Fig. S3 Expression profiles of C-type lectin-like genes in Lb larval secretions
